# Relationship Between CRISPR–Cas Systems and Acquisition of Tetracycline Resistance in Non-Clinical *Enterococcus* Populations in Bulgaria

**DOI:** 10.3390/antibiotics14020145

**Published:** 2025-02-02

**Authors:** Maria Pandova, Yoana Kizheva, Petya Hristova

**Affiliations:** Department of General and Industrial Microbiology, Faculty of Biology, Sofia University, 1164 Sofia, Bulgaria; maria.pandova@abv.bg (M.P.); pkabad@biofac.uni-sofia.bg (P.H.)

**Keywords:** CRISPR–Cas systems, antibiotic resistance, tetracycline, horizontal gene transfer, enterococci

## Abstract

Non-clinical enterococci are relatively poorly studied by means of acquired antibiotic resistance to tetracycline and by the distribution, functionality and role of their CRISPR systems. **Background:** In our study, 72 enterococcal strains, isolated from various non-clinical origins, were investigated for their phenotypic and genotypic (*tet*(M), *tet*(O), *tet*(S), *tet*(L), *tet*(K), *tet*(T) and *tet*(W)) tetracycline resistance. **Methods:** The genetic determinants for HGT (MGEs (*Int-Tn* and *prg*W), inducible pheromones (*cpd*, *cop* and *cff*), aggregation substances (*agg*, *asa1*, *prgB* and *asa373*) and CRISPR–Cas systems were characterized by PCR and whole-genome sequencing. **Results:** Four *tet* genes (*tetM*, *tetO*, *tetS* and *tetT*) were detected in 39% (n = 28) of our enterococcal population, with *tet*M (31%) being dominant. The gene location was linked to the Tn6009 transposon. All strains that contained *tet* genes also had genes for HGT. No *tet* genes were found in *E. casseliflavus* and *E. gilvus*. In our study, 79% of all *tet*-positive strains correlated with non-functional CRISPR systems. The strain *E. faecalis* BM15 was the only one containing a combination of a functional CRISPR system (*cas1*, *cas2*, *csn2* and *csn*1/*cas*9) and *tet* genes. The CRISPR subtype repeats II-A, III-B, IV-A2 and VI-B1 were identified among *E. faecalis* strains (CM4-II-A, III-B and VI-B1; BM5-IV-A2, II-A and III-B; BM12 and BM15-II-A). The subtype II-A was the most present. These repeats enclosed a great number of spacers (1–10 spacers) with lengths of 31 to 36 bp. One CRISPR locus was identified in plasmid (p.Firmicutes1 in strain *E. faecalis* BM5). We described the presence of CRISPR loci in the species *E. pseudoavium*, *E. pallens* and *E. devriesei* and their lack in *E. gilvus*, *E. malodoratus* and *E. mundtii*. **Conclusions:** Our findings generally describe the acquisition of foreign DNA as a consequence of CRISPR inactivation, and self-targeting spacers as the main cause.

## 1. Introduction

*Enterococcus* species are known to be spread in various environments (gastrointestinal tracts (GITs) of mammalian, avian and invertebrate species as well as natural habitats such as water, soil, plants, etc.) [1]. Generally, enterococci are Gram-positive, non-motile, catalase-negative bacteria and facultative anaerobes. Currently, approximately 60 enterococcal species are published with valid names [2]. Many cases describing enterococci as leading causative agents of hospital-acquired infection (bacteremia, urinary tract infection (UTI), endocarditis, etc.) have been reported, with *Enterococcus faecalis* and *Enterococcus faecium* being the most commonly associated with this [3,4]. The successful treatment of such infections is a challenge due to the widespread antibiotic resistance among these bacteria [5]. Interestingly, genetic determinants for antibiotic resistance, specifically for tetracycline, have been described in the literature for species other than *E. faecium* and *E. faecalis* (e.g., *Enterococcus hirae* and *Enterococcus casseliflavus*) [6]. It is known that enterococci are part of the plant microbiome from where they enter the intestinal tracts of animals and humans through the intake of plant food. Each gut microbiome selects the enterococcal species it needs to maintain eubiosis. Colonizing different microbiomes, from invertebrates to mammals, allows plant enterococci to acquire new genes, which they then spread into new environmental niches. This life cycle of passing through hosts from different biological kingdoms defines enterococci as important vectors for the horizontal transfer of antibiotic resistance and virulence genes, despite where they originate from. Moreover, the remarkable ability of enterococci to acquire and transfer antibiotic resistance genes through horizontal gene transfer (HGT) is another concerning issue. This transfer of antibiotic resistance genes is mediated mainly by mobile genetic elements (MGEs) like plasmids, phages and transposons [5]. Genetic virulence determinants are also described to be transferred trough plasmids and/or transposons. The pheromone-responsive plasmids pAD1 and pCF10, for example, are known to contain cytolysin and aggregation substances [5]. These biofilm formation-responsible substances can have a role in the gene transfer processes as well. They can contribute to the spatial arrangement and close proximity of the enterococcal strains, which can facilitate easier gene transfer between different strains. In addition, the products of pheromone-encoding genes are involved in initiating characterized conjugation processes. They are also thought to be involved in eliciting an inflammatory response [7].

It is assumed that a limiting factor in the development of antibiotic resistance in enterococci is Clustered Regularly Interspaced Short Palindromic Repeats (CRISPR)–Cas systems [8]. The mechanism of action of those systems is known to be the recognition and cleavage of foreign nucleic acids in three consecutive phases: adaptation (acquisition), expression (biogenesis) and interference [9]. These systems are composed of CRISPR arrays and CRISPR-associated (Cas) proteins, and represent an adaptive immune system in bacteria that is an analog of the immune systems of mammalians [10]. CRISPR arrays consist of short, repetitive DNA sequences and unique spacer sequences (protospacers) derived from foreign genetic material (phage, plasmids and transposons). Although the main role of these systems is related to the development of phage resistance, some studies have reported their role in conferring immunity to plasmids as well. Moreover, an inverse correlation between the possession of complete CRISPR loci and antibiotic resistance has been found [11]. The most frequently reported data concern the distribution of CRISPR–Cas systems and discuss this relationship primarily among clinically associated enterococcal isolates, mainly *E. faecalis* [12].

Generally, based on the effector Cas proteins, CRISPR–Cas systems are classified into two classes: class 1 (type I, III and IV) and class 2 (type II, V and VI). Additionally, 33 subtypes have also been described. The presence of Cas-associated proteins defines the functionality of the CRISPR–Cas systems; therefore, functional CRISPR–Cas systems are less distributed among multidrug-resistant bacteria, particularly enterococci. In this regard, the major Cas-associated proteins for class 1 CRISPR–Cas systems are listed as Cas3 (for type I) and Cas10 (for type III), while for class 2 these are Cas9 (for type II), Cas12 (for type V) and Cas13 (for type VI) [9]. The most prevalent CRISPR–Cas system among enterococci is type II-A with three loci: CRISPR1-Cas, CRISPR2 (orphan locus, usually lacking *cas* genes) and CRISPR3-Cas [12].

In contrast to intrinsic antibiotic resistance in enterococcus species (to beta-lactams, glycopeptides and fluoroquinolones, with high-level resistance to aminoglycosides), resistance to tetracycline, as well as to other antibiotics like erythromycin, can be a result of HGT in these bacteria [13]. In this regard, the wide use of tetracyclines as a food additive in livestock breeding (incl. snail farms, cow farms, poultry farms, etc.) represents a hazard due to the possibility of the genes encoding tetracycline resistance to be transferred to humans through the food chain [14]. Generally, two major groups of *tet* genes have been identified in enterococci. The first group is connected with the energy-dependent efflux of tetracycline from the cells (*tet*(K) and *tet*(L)), while the second group includes genes encoding resistance by ribosomal protection (*tet*(M), *tet*(O), *tet*(S), *tet*(T) and *tet*(W)) [15]. A common mechanism for the HGT of tetracycline resistance genes is known to be through MGEs (phages, plasmids and transposons) [16]. In a recently published study, a distribution of *tet* genes among an enterococcal population isolated from poultry feces in Nigeria has been demonstrated. Interestingly, the *tet*(M) gene was found predominantly in *E. casseliflavus* isolates instead of in *E. faecalis* [17]. Moreover, the most prevalent gene encoding tetracycline resistance (*tetM*) in enterococci was found to be connected to the conjugative transposon Tn916 [17]. Analyses of the bacterial genomes imply a connection between CRISPR–Cas systems and the MGEs [12].

Except for several recently published papers, there are still insufficient data on the distribution, functionality and role of CRISPR–Cas systems and antibiotic resistance to tetracycline among enterococcal species of non-clinical origin [14,17,18]. In our previous study, we described the distribution of pathogenic potential among non-clinical enterococci in various sources (dairy and meat products, GITs of herbivores and human breast milk) [19]. Thus, as a continuation, in this study, we aimed to investigate the relationship between CRISPR–Cas systems and the acquisition of tetracycline resistance in enterococcus species isolated from animal dairy products (young and mature feta cheese, cow milk and yogurt), the GITs of invertebrates (*Cornu aspersum*), ready-to-eat food containing meat (Doner kebab) and human breast milk.

## 2. Results

The objects of this study were 72 enterococcal strains, isolated from various environmental niches (Bulgarian yogurt, GIT of *C. aspersum*, young and mature feta cheese, sow milk, Doner kebab and human breast milk). Among the tested strains, the following species from the genus *Enterococcus* were used: *Enterococcus* sp. (n = 2), *E. mundtii* (n = 6), *E. casseliflavus* (n = 8), *E. gilvus* (n = 1), *E. pseudoavium* (n = 1), *E. pallens* (n = 1), *E. malodoratus* (n = 1), *E. devriesei* (n = 2), *E. gallinarum* (n = 3), *E. durans* (n = 5), *E. faecium* (n = 10) and *E. faecalis* (n = 32) [19].

### 2.1. Phenotypic and Genotypic Tetracycline Resistance

The phenotypic analysis for tetracycline resistance showed large variability in the inhibition zones: from 13 mm to 50 mm. According to CLSI standards, 16 (22%) of all tested strains showed resistance to tetracycline and formed zones ≤ 14 mm. The majority of these strains belong to the species *E. faecalis* isolated from human breast milk and young feta cheese. Single representatives of *E. gallinarum* (BY17), *Enterococcus* sp. (BY8), *E. casseliflavus* (BY9) and *E. pseudoavium* (CA9), isolated from Bulgarian yogurt and the GIT of *C. aspersum*, respectively, were also found to be resistant to tetracycline. The percentage of strains with intermediate sensitivity is 8% (6 strains), while 70% are sensitive (50 strains) (Table 1 and Table S1).

**Figure 1:**
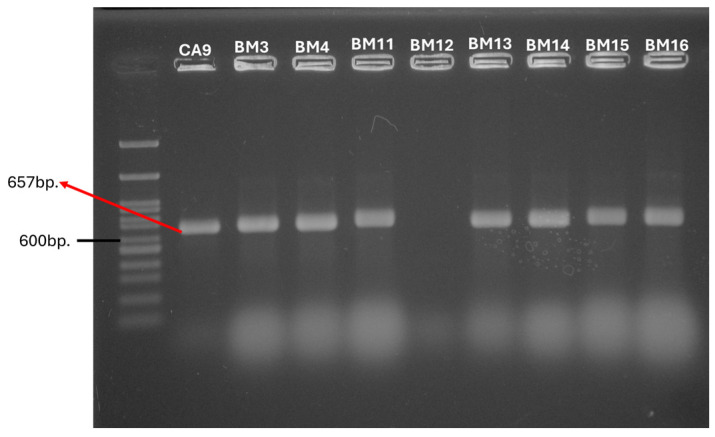
PCR amplification of the *tetM* gene.

To prove the phenotypic tetracycline resistance, the corresponding genetic determinants were screened. The results showed that out of seven tested *tet* genes, a positive amplification product was only obtained for four of them (*tetM*, *tetO*, *tetS* and *tetT*) in 39% (n = 28) of all tested strains. The remaining 44 strains (61%) were *tet*-negative. The most distributed gene for tetracycline resistance was *tetM*, found in 31% (n = 22) of all tested strains (Figure 1, Table 1).

The genes *tetS*, *tetO* and *tetT* were less represented among the investigated enterococcal populations: 8% (n = 6; *E. faecalis* strain YFC3 and BM15, *E. mundtii* strain CA8, *E. pallens* strain CA10 and *E. devriesei* strains CA13 and CA16), 3% (n = 2; *E. durans* strain YFC5 and *E. malodoratus* strain CA11) and 1% (n = 1; *E. gallinarum* strain CA15), respectively. In the analyzed populations, no strains containing *tetK*, *tetL* or *tetW* were detected (Table S1).

Comparison of the positive PCR amplification for *tet* genes and the size of the inhibition zones showed that the *tetT*-positive strain (CA15) gave an inhibition zone of 35 mm. The two *tetO*-positive strains (YFC5 and CA11) showed 35 mm and 50 mm zones, respectively. Most of the strains with phenotypic tetracycline resistance contain predominantly the *tetM* gene (n = 14). However, in three strains (*E. faecalis* YFC3, *E. pallens* CA10 and *E. faecalis* BM15) we found a combination of *tetM* and *tetS* genes. Two of these strains (YFC3 and CA10) showed strong and intermediate susceptibility, respectively.

**Figure 2:**
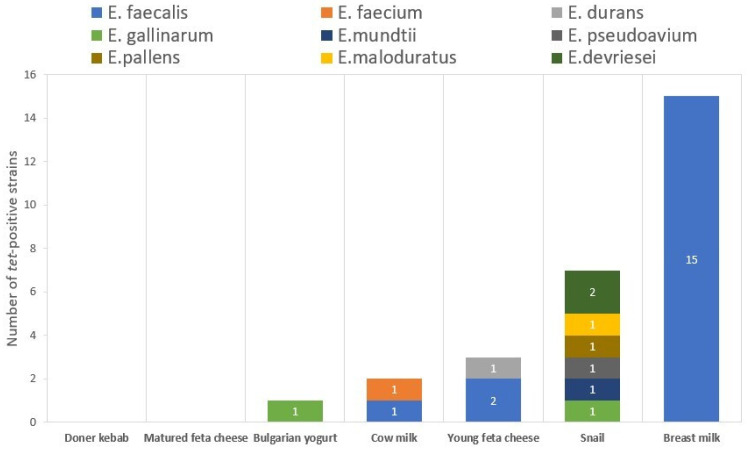
Distribution of *tet*-positive strains across the analyzed origins.

The monitoring of the distribution of *tet* genes among the isolates with different origins shows that the isolates with the most tetracycline resistance genes are from breast milk (n = 15), followed by the isolates from the GIT of *C. aspersum* (n = 7) and those from young feta cheese (n = 3). The food with the lowest number of *tet*-positive strains was the Bulgarian yogurt (1 out of 27 strains). No genes for tetracycline resistance were found in the Doner kebab isolate, as well as in the two isolates from mature feta cheese. No tetracycline resistance genes were found in the *E. gilvus* and *E. casseliflavus* isolates (Figure 2).

The statistical analysis revealed significant differences between the number of tetracycline resistance genes in *E. faecalis* and non-*E. faecalis* species (*p* < 0.05). No significant differences were established between the number of tetracycline resistance genes in the food and *C. aspersum* isolates (*p* > 0.05). However, the differences were significant between the food and breast milk strains (*p* < 0.05), as well as between the *C. aspersum* and breast milk isolates (*p* < 0.05).

### 2.2. Detection of CRISPR–Cas Loci

All strains were tested for the presence of CRISPR loci and their associated Cas proteins by conventional PCR. Our results showed that 42% (n = 30) of all tested strains had at least one CRISPR locus in their genomes (Table 2).

Almost half of them belonged to the breast milk isolate group (47% of all CRISPR-positive strains, n = 14). Another 37% (n = 11) of them were isolated from food sources, followed by 17% (n = 5) which came from the intestinal tract of snails. Out of the three analyzed types of CRISPR loci, the most abundant was the orphan CRISPR2 type (n = 27; 79% of all positive strains). CRISPR1 loci were amplified in 67% of all positive strains (n = 20). The least amplified was the CRISPR3 locus, presented in 41% of all positive strains. Only one of our strains showed amplification for the *csn*1 (later described as *cas*9) gene (*E. faecalis* BM15).

The analysis of the distribution of CRISPR systems among the different enterococcal species showed that from all positive strains, 60% (n = 18) were assigned to the *E. faecalis* species. The rest were distributed as follows: 13% (n = 4) *E. casseliflavis*; 7% (n = 2) *E. faecium* (CM1 and DK1) and *E. durans* (YFC2 and YFC4); and 3% (n = 1) *E. pseudoavium* (CA9), *E. pallens* (CA10), *E. devriesei* (CA13) and *E. gallinarum* (CA15). Also, 79% (n = 22) of all *tet*-positive strains gave amplification for CRISPR loci.

According to the origin of the strains, four out the five CRISPR-positive isolates from *C. aspersum* have mostly CRISPR2 systems, and only one of them (*E. pallens* CA10) has a combination of two systems (CRISPR2 and CRISPR1). Among the *E. faecalis* strains isolated from breast milk (n = 16), 87% (n = 14) were positive for CRISPR loci and two isolates (13%) were negative. Interestingly, 71% (n = 10) of them had all three types of CRISPR loci; 21% (n = 3) had a combination of CRISPR2 and CRISPR1. Only one of them (*E. faecalis* BM15) displayed a positive PCR amplification for the CRISPR2 locus. That was also the only strain that showed amplification for the *csn1* (later described as *cas9*) gene.

The strains isolated from food (n = 39) also showed diversity in the type of the detected CRISPR systems. Of these, 28% (n = 11) showed positive amplification for CRISPR loci. Three isolates show a positive profile only for CRISPR2 (*E. faecium* DK1 and *E. casseliflavus* BY20 and BY21). The rest of the eight positive strains showed different combinations of the three types of CRISPR loci. One of them had the combination CRISPR1 + CRISPR2 (*E. casseliflavus* BY18), two strains had combination of CRISPR2 + CRISPR 3 (*E. durans* YFC2 and YFC4) and another two strains had the combination of CRISPR1 + CRISPR2 + CRISPR3 (*E. faecium* CM1 and *E. faecalis* CM4). Three strains contained only the CRISPR1 locus (*E. faecalis* YFC1, YFC3 and BY22). No CRISPR loci were detected in the species *E. gilvus*, *E. malodoratus* and *E. mundtii*.

The statistical analysis showed significant differences in the number of CRISPR loci only between the breast milk and food isolates (*p* < 0.05), as well as between the breast milk and snail isolates (*p* < 0.05). There were no significant differences in the number of CRISPR loci between the food and snail isolates (*p* > 0.05). Significant differences were established between the number of CRISPR loci detected in *E. faecalis* and other enterococcal species (*p* < 0.05).

### 2.3. PCR Detection of the Genetic Determinants for HGT

#### 2.3.1. Aggregation Substances

The strains were tested for the presence of four aggregation substance genes—*agg*, *asa1*, *prgB* and *asa373.* Positive amplifications were established for 39% (n = 28) of all strains (Table 3).

Of these, 71% (n = 20) belonged to *E. faecalis* species. The remaining eight strains belong to the species *E. faecium* (n = 6; CM1 and BY12-BY16), *E. devriesei* (n = 1; CA13) and *E. mundtii* (n = 1; CA8). Positive amplifications were established for two strains, isolated from the GIT of *C. aspersum* (*E. mundtii* CA8 and *E. devriesei* CA13); 14 strains from breast milk; and 12 strains from food. The gene *prgB* was the most amplified one among the tested strains (75% of all positive strains, n = 21), followed by *asa1* with 68% (n = 19), *agg* with 39% (n = 11) and *asa373* with 4% (n = 1; *E. faecalis* BY11). The statistical analysis showed no significant difference in the number of aggregation substance genes between the food and snail isolates (*p* > 0.05). On the other hand, there were significant differences between the food and breast milk isolates (*p* < 0.05) and between the snail and breast milk isolates (*p* < 0.05). The analysis also showed significant differences in the number of aggregation substance genes between *E. faecalis* strains and the other species (*p* < 0.05).

#### 2.3.2. Inducible Pheromones

The analyzed strains were amplified with primers for three inducible pheromone genes—*cpd*, *cop* and *cff*. Positive amplifications were detected for 76% (n = 55) of all tested strains (Table 3). The predominant gene in all positive isolates was *ccf* with 90% (n = 50), followed by *cpd* with 65% (n = 36) and *cob* with 36% (n = 20). The screened genes were present in a wide range of species, but the majority of sex pheromone-positive strains belong to *E. faecalis* (56% of all positive strains, n = 31). However, positive strains were found within the following species: *E. faecium* (16%, n = 9), *E. durans* (4%, n = 2), *Enterococcus* sp. (4%, n = 2), *E. casseliflavus* (9%, n = 5), *E. gallinarum* (5%, n = 3), *E. mundtii* (2%, n = 1) and *E. pallens* (2%, n = 1). The statistical analysis also showed significant differences in the number of sex pheromone genes between *E. faecalis* and the other enterococcal species (*p* < 0.05).

As for the origin of the strains, all of those isolated from breast milk (n = 16) and the majority of the food isolates (87%, n = 34) were positive. Only five strains (29%) of the snail isolates generated the searched fragments for these genes. The statistics showed that there were significant differences between the number of sex pheromones in all three groups—food and snail isolates (*p* < 0.05); food and breast milk isolates (*p* < 0.05); and snail and breast milk isolates (*p* < 0.05).

#### 2.3.3. MGEs

The presence of the *Tn916–1545* transposon family and the pheromone-responsive plasmid *pCF10* were determined by amplifying *Int-Tn* (the integrase gene for the *Tn916–1545* transposon family) and *prg*W (replication initiator protein gene of *pCF10*), respectively. The obtained results showed that 19% (n = 14) of all strains tested were positive for the integrase gene, while 22% (n = 16) generated the fragment for *prg*W (Table 3). All of the strains harboring these MGEs were representatives of the *E. faecalis* species. The majority of them were isolated from human breast milk (81% of all positive strains, n = 13). Only three strains (CM4, YFC1 and YFC3) were part of the food group. The isolates from *C. aspersum* did not carry genes for MGEs. Most of the strains that had the *Tn916–1545* transposon also harbored the *pCF10* plasmid in their genomes. The exceptions are two strains *E. faecalis*—BM13 (*prg*W (+), *Int-Tn* (−)) and BM14 (*prg*W (−), *Int-Tn* (+)).

### 2.4. Bioinformatic Analyses of E. faecalis Genomes

The genomes of the four *E. faecalis* strains (CM4, BM5, BM12 and BM15) were obtained with more than 100× coverage. All bacterial chromosomes were full with circular topology (Supplementary Figures S1–S4). All of the strains have chromosomes about 2.9 Mb in length and a GC content of approximately 37% (CM4–37.49%; BM5–37.46%; BM12–37.7%; BM15–37.38%). The processed sequences were deposited at NCBI Genome with approved accession numbers as follows: CP173761-CP173764 (*E. faecalis* CM4); CP173669-CP173671 (*E. faecalis* BM5); CP173666-CP173668 (*E. faecalis* BM12); CP173758-CP173760 (*E. faecalis* BM15).

**Figure 3:**
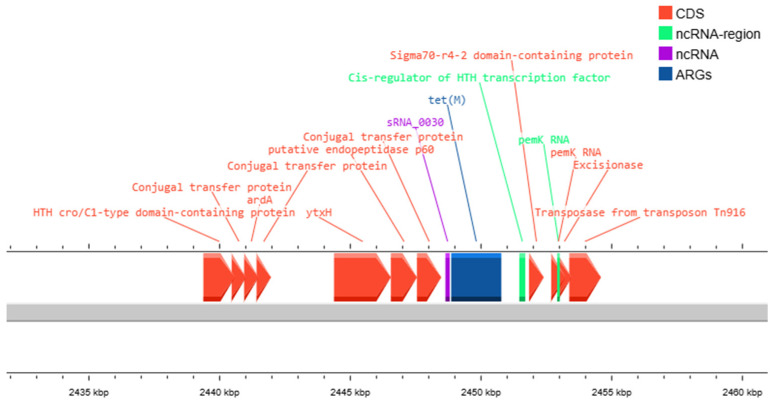
Localization of *tetM* gene in close proximity to transposable elements. Gene for Tn916 transposase can be seen in this annotated DNA sequence. Legend: CDS-coding sequence; ncRNA- (cis-regulatory) region (non-coding RNA region); ncRNA (non-coding RNA); ARGs—antibiotic resistance genes. The image was generated with Map Builder in Proksee software, version 2.0.5. (https://proksee.ca/, accessed on 13 November 2024).

Annotation by CARD (Comprehensive Antibiotic Resistance Database) in Proksee for tetracycline resistance genes confirmed the PCR results. All examined *E. faecalis* strains (CM4, BM5, BM12 and BM15) contained *tet* genes in their genomes. As per the PCR results indicated, CM4, BM5 and BM12 had only the *tetM* gene in their genomes. BM15, as detected by PCR amplification, had two tetracycline resistance genes—*tetM* and *tetS*. Moreover, the localization of *tetM* in the genomes of our isolates was found to be near the Tn916 transposone (Figure 3).

The bioinformatical analyses confirmed the PCR results for the presence of CRISPR loci in the tested strains, as well as the *cas* gene (*csn*1/*cas*9) in only one strain (*E. faecalis* BM15). Moreover, three additional *cas* genes were found in this strain (*cas1*, *cas2* and *csn2*). After whole-genome sequencing of our strains, two to three CRISPR loci were identified, the majority of which localized in the bacterial chromosome. The CRISPR systems, localized in the chromosome or in plasmid, were identified by subtyping the CRISPR repeats. Using this method, in our isolates four different types of CRISPR systems were identified: II-A, III-B, IV-A2 and VI-B1 (Table 4). The CRISPR II-A repeats were found in the genomes of all of the analyzed strains, which makes them the most widely distributed systems across the analyzed strains in our study. These repeats enclosed a great number of spacers (1–10 spacers) with lengths of 31 to 36 bp. The identified targets for these spacers were part of phages’ genomes or the *E. faecalis* chromosome. Strain *E. faecalis* CM4 containthree types of CRISPR repeats (II-A, III-B, VI-B1). Strain *E. faecalis* BM5 also contains three types (IV-A2, II-A and III-B). Strains *E. faecalis* BM12 and BM15 have only the II-A type repeat.

**Figure 4:**
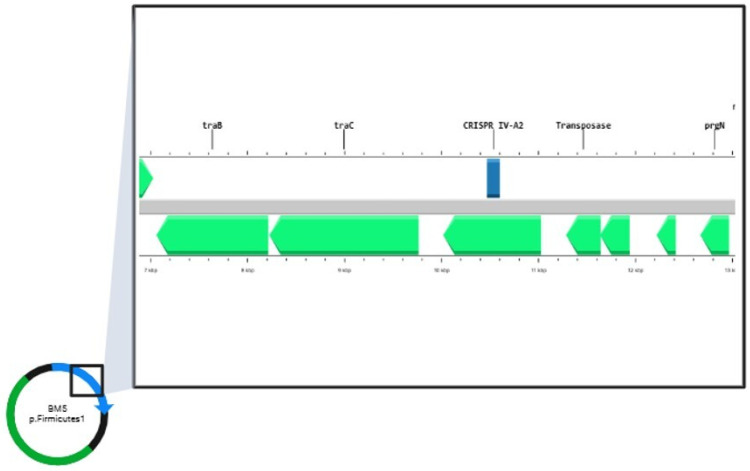
Localization of CRYSPR-Cas system type IV-A2 on plasmid p.Firmicutes1 in strain *E. faecalis* BM5. The image was generated with Map Builder in Proksee software, version 2.0.5. (https://proksee.ca/, accessed on 13 November 2024).

The identified targets in the aforementioned CRISPR loci are part of phage genomes, chromosomes of *E. faecalis* strains or plasmids (Supplementary Figures S1–S4). Target analysis of the CRISPR–Cas systems in the plasmid database (RefSeq-Plasmid) showed that the repeat, categorized as part of the IV-A2 CRISPR system in *E. faecalis* BM5 was found to be localized on plasmid p.Firmicutes1 (Figure 4).

This was the only CRISPR–Cas system targeting plasmids in our study. After annotation of the plasmid sequence, it was found that this CRISPR system is near transposable elements (*tra* genes, transposases and resolvases) (Figure 4).

The RNA secondary structures and minimum free energy (MFE) of the repeat sequences were predicted using the RNAfold web server. The MFE value ranged from −7.10 kcal/mol to −1.4 kcal/mol. The secondary RNA structure with the lowest MFE is part of the IV-A2 CRISPR system, located on the plasmid of *E. faecalis* BM5 (Table 4).

Interestingly, all sequenced strains (including the only strain with functional a CRISPR system, *E. faecalis* BM15) contained plasmids in their genomes (Supplementary Figures S1–S4). In each strain, two to three plasmid sequences were detected. The plasmid size varied from 5 kB (p.Firmicutes2 in CM4) to 113 kB (p.Firmicutes1 in CM4). In each sequenced *E. faecalis* strain, at least one pheromone-responsive conjugative plasmid was detected (p.Firmicutes1 in BM5 and CM4, p.Firmicutes2 in BM12 and BM15). In each of them, *prg* genes, found in pheromone-responsive plasmids, were detected. Although there were plasmids in the strains’ genomes, only one of them (p.Firmicutes1 in *E. faecalis* BM15) contained antibiotic resistance genes (*erm*B, *aad*(*6*), SAT-4, CAT). The absence of tetM genes on plasmid sequences directed the study to the annotation of other mobile genetic elements in our sequences. As a result, the mutual conjugative transposable element Tn6009 was found. Moreover, we established that the *tet*M genes, found in our strains, were located next to this transposon (19220–19230 bases upstream or downstream from this element).

## 3. Discussion

In this study, 72 non-clinical enterococcal strains, isolated from different ecological setting (food products, human breast milk and the GIT of *C. aspersum*), were investigated. The main aim of the paper is to study the relationship between tetracycline antibiotic resistance, the presence of MGEs and HGT determinants, and CRISPR loci in relatively poorly studied enterococci, different from *E. faecalis* and *E. faecium*.

Tetracycline resistance genes were detected in 39% of our non-clinical enterococcal strains. This shows that tetracycline resistance genes are distributed in the analyzed enterococcal population in Bulgaria. Analysis of the antibiotic resistance in the microbiota of wastewater from Bulgaria, published in 2024, shows similar results with the presence of tetracycline-resistant strains in more than 35% of the analyzed strains [20]. Another study on clinical enterococci in Bulgaria describe tetracycline resistance in 69% of the analyzed strains [21]. Overall, the tetracycline resistance in enterococci globally is shown to increase over time [22], which once more underlies the importance of its surveillance. Moreover, a significant number of tetracycline resistance genes have already been dominant in clinical enterococcal isolates even in the early years of use of tetracycline [15]. In our study, the dominant tetracycline resistance gene was *tetM*, similarly to other enterococcal populations isolated from different ecological niches [20,23,24]. The established strong presence of *tetM* genes in the breast milk isolates (in 94% of the strains) suggests its role in the newborn gut resistome. The presence of antibiotic resistance genes in the microbiome of breast milk suggests their presence in the infant resistome. Similar suggestions, reporting a shared resistome and mobile genetic elements in the microbiome of newborn feces and the breast milk of their mothers, have been reported in the literature [24]. Other sources suggest a vaginal origin of *tetM* genes in newborns born via vaginal delivery [25]. These data suggest that antibiotic resistance genes in the microbiota of newborns may enter through different routes—via nutrition and during the birth process. That could explain the wide abundance of tetracycline resistance in mammals, starting with their birth and continuing with their feeding, both as newborns and in their sources of food as grownups.

The presence of *tet* genes in this study, however, was relatively low in enterococcal species isolated from food (Bulgarian yogurt, cow milk and feta cheese), which did not completely support the other published data [23]. Only 6 out of 39 (2.5%) of our strains, isolated from food, had tetracycline resistance genes. Investigation of different Belgian foods showed that non-clinical isolates of *E. faecalis* (71%), *E. faecium* (6.6%), *E. durans* (15%) and *E. gallinarum* (2.2%) were found to carry *tet*(M) [15]. As described in the literature, *E. faecalis* most frequently harbors tetracycline resistance genes [26]. In our work, we did not find *tet* genes in *E. casseliflavus* and *E. gilvus* strains, similarly to other studies [15]. Although poorly described, some authors mention the lack of *tet* genes in *E. casseliflavus* or an incapability of the gene expression, while others describe the opposite [17,27].

The comparison between the PCR amplification for *tet* genes and the size of the inhibition zones shows that not all of the *tet* genes (*tetT* and *tetO*) are functionally active. The inhibition zones show that strains containing those genes are susceptible to tetracycline, which leads one to the assumption that these genes are not expressed in the tested conditions. In addition, we observed that most of the strains showing phenotypic tetracycline resistance contain *tetM* gene in their genomes, which we assumed to be due to the expression of the *tetM* gene. Moreover, the *tetM* gene location was linked to the *Tn6009* transposon. That transposon is part of the Tn916 family and was previously described in the literature to be linked to *tetM* genes [28]. Moreover, all strains that contain MGEs also had *tet* genes (and more specifically the *tetM* gene). All of them also amplified genes for sex pheromones and aggregation substances. The presence of *prgW* correlated with the presence of the *asa1* (gene for aggregation substance) gene in all positive strains.

CRISPR systems are widely described in the literature as interfering with phage infections and, on the other hand, as mechanisms which are building a barrier for horizontal gene transfer, as these systems could disable the acquisition of plasmids and other exogenic DNA [29]. All tested strains (except *E. faecalis* BM15) in our study lacked or had non-functional (had no *cas* genes) CRISPR systems. Some authors explain the loss of *cas* genes as a mechanism of multidrug-resistant bacteria to maintain antibiotic resistance genes, valuable for their survival [30]. In our study 79% of all *tet*-positive strains correlated with non-functional CRISPR systems, which could be explained as a result of the self-inactivation of these systems to maintain the *tet* genes. However, the strain with the only functional CRISPR system found in this study (*E. faecalis* BM15) also contained *tet* genes. Some authors describe a lack of functionality of CRISPR systems due to mutations in the promoters of the *cas* genes [31]. On the other hand, data revealing a positive correlation between functional CRISPR systems and the presence of antibiotic resistance genes can also be found [11]. Another inconsistency with this theory is that in the sequenced genomes only one strain (*E. faecalis* BM5) had spacer matching with plasmids. Therefore, the inactivation of the CRISPR systems, in order to maintain plasmids in the cell, cannot be valid in this case. The majority of the spacers aligned with phage DNA material. Another part of the spacers targeted the bacterial self-genome. Bacterial DNA-targeting spacers are found in the literature as well [32]. Some authors describe self-targeting spacers as a type of gene regulatory systems [33]. Other authors describe this type of self-targeting as part of autoimmunity in bacteria. This type of interaction is linked with the partial or full degradation of CRISPR activity [34]. In our study, as in reference [35], CRISPR3 loci were the least amplified among the studied enterococcal strains.

The information in the literature about the distribution of CRISPR systems is mostly focused on their prevalence in clinical isolates of *E. faecalis* and *E. faecium* strains [12]. Scarce data are available about the dissemination of these adaptive systems in other enterococcal species, even more so regarding non-clinical origins. The distribution of CRISPR–Cas systems may vary by species due to the different selective pressures exerted by the different environments of enterococcal species [36]. Here, we describe the lack of CRISPR loci in the species *E. gilvus* (n = 1), *E. malodoratus* (n = 1) and *E. mundtii* (n = 6). We also describe the presence of CRISPR loci in the species *E. pseudoavium, E. pallens* and *E. devriesei*, which, to our knowledge, are not to this date mentioned in CRISPR studies. Similarly, among 110 investigated non-clinical enterococcal strains, 517 different CRISPR spacers were detected in species other than faecium and faecalis (*E. avium* (n = 2), *Enterococcus cecorum* (n = 3), *E. durans* (n = 6), *E. hirae* (n = 10), *E. mundtii* (n = 6), *Enterococcus silesiacus* (n = 1), *Enteroccus thailandicus* (n = 1) and *Enterococcus* sp. (n = 5)) [11].

The CRISPR repeats in the sequenced strains were typed as four different subtypes (II-A, VI-B1, III-B and IV-A2). A repeat, which was subtyped as II-A, was present in all of the sequenced strains. This subtype of a direct repeat sequence was also commonly found in other works [32]. One CRISPR locus was also identified in plasmid (p.Firmicutes1 in strain *E. faecalis* BM5). This is an interesting finding considering the evolutions and distribution of the CRISPR systems. According to the literature, defective variants lose their adaptive and interference functions and gain roles different from the adaptive immunity of bacteria. These types of CRISPR variants are localized on transposons and plasmids [37]. The system, located on a plasmid in our study, was found to be close to transposases, resolvases and genes responsible for the non-sexual transfer of genetic elements in the genome. Similar findings have been reported in the literature as one of the most described subtypes of CRISPR systems was I-F, located next to the big transposon family Tn7 [38]. Redando et al. [39] also describe that prokaryotic mobile genetic elements (mostly plasmids) are primarily responsible for encoding type IV CRISPR–Cas system loci. They suggest that, in order to dominate the host environment, plasmid-like elements use type IV systems to eradicate other plasmids with comparable characteristics. The bioinformatic analyses with RepeatTyper identified the repeat in the plasmid of *E. faecalis* BM5 as part of the IV-A2 system. All secondary RNA structures of the repeats formed stem–loop structures with low minimum free energy, which indicates the stability of these structures.

A strong connection between the presence of tetracycline resistance genes and genetic determinants for HGT was established in our study. This, as well as the inactive CRISPR systems, shows the potential of these strains to acquire mobile genetic elements (for example, virulence and tetracycline resistance genes). The potential of genetic transfer by the presence of aggregation substances and sex pheromones was presented best in *E. faecalis* strains, isolated from human breast milk. *E. faecalis* is a species widely described in the literature as the species with the most potential to cause infections in humans and as one of the main causatives of nosocomial infections [40]. In our study, the potential for acquiring mobile genetic elements was further confirmed by the presence of plasmids and transposable elements. Another important correlation can be seen in this study—the presence of *prgW* correlated with the presence of *asa1*. This aggregation substance gene may be one of the main factors determining the transfer of the pheromone-inducible plasmids. All of the analyzed strains carried more than one plasmid in their genomes. That is another confirmation of the inactive CRISPR systems, although spacers targeting plasmids were not detected in all of the strains. That means that the plasmid acquisition could be a consequence of the system inactivation, but not its reason.

## 4. Materials and Methods

### 4.1. Bacterial Strains, Growth Conditions and DNA Isolation

A total of 72 previously identified enterococcal strains isolated from milk and meat food products (n = 39), the GIT of *C. aspersum* (n = 17) and from breast milk (n = 16) were used in this study [19]. All strains were cultivated on de Man, Rogosa and Sharpe (MRS) broth (Merck, Darmstadt, Germany) for 24 h at 37 °C. The resulting enriched cultures were used for total DNA isolation using a GenElute Bacterial Genomic DNA Kit, according to the manufacturer recommendations, with slight modifications (Sigma-Aldrich Co., 3050 Spruce Street, St. Louis, MO 63103, USA). For more effective cell lysis, 2 μL (1000 units/mg) mutanolysin (Merck KGaA, Darmstadt, Germany) was added. The quality and quantity of the obtained DNAs were checked electrophoretically on 1% agarose gel and on spectrophotometer/fluorometer DeNovix DS-11 FX+ (DeNovix Inc., Wilmington, DE, USA), respectively. The purified DNAs were stored at −20 °C for further analysis.

### 4.2. Phenotypic Determination of Tetracycline Resistance

Phenotypic susceptibility to tetracycline (30 µg/disc) was investigated in accordance with the Kirby–Bauer disc diffusion method [41]. Log-phase bacterial cultures with approximate concentrations of 10^8^ CFU/mL (MacFarland units 0.5) were prepared in MRS broth (16 h at 37 °C). Aliquots of 0.1 mL bacterial cultures were poured into 20 mL of Muller Hinton agar (Merck KGaA, Darmstadt, Germany) in Petri dishes. The plates were incubated at 37 °C for 24 h. The diameters of the inhibition zones were measured, and the strains were classified as resistant (≤14 mm, R), strong susceptible (≥19 mm, S) and intermediate susceptible (15–18 mm, I), according to CLSI M100-Ed34, 2024 [42].

### 4.3. PCR-Based Methods

All PCR amplifications were performed in a total reaction volume of 25 µL, containing 16.5 μL ultrapure H_2_O, 0.5 μL (5 pmol/μL) of each primer, 6.5 μL VWR Red Taq polymerase master Mix (VWR International bvba/sprl, Haasrode Researchpark Zone 3, Geldenaaksebaan 464 B-3001, Haasrode Belgium) and 1 μL of template DNA. The PCR products were analyzed by electrophoresis using a 1.5% agarose gel in 1X TBE buffer at 100 V for 30 min. For the size evaluation of the amplified products, a 100 bp DNA ladder (SERVA FastLoad 100 bp DNA ladder, SERVA Electrophoresis GmbH, Carl-Benz-Str. 7, Heidelberg, Germany) molecular size marker was used.

#### 4.3.1. Detection of Tetracycline Resistance Genes

Genotypic resistance to tetracycline was evaluated via the detection of antibiotic resistance genes, using polymerase chain reaction (PCR). Seven different genes (*tetM*, *tetK*, *tetL*, *tetO*, *tetS*, *tetW* and *tetT*), encoding tetracycline resistance, were screened according to Aarestrup et al. [14], Aminov et al. [43] and Doherty et al. [7]. The reactions conditions were as follows: initial denaturation at 94 °C for 10 min, followed by 30 cycles of denaturation at 94 °C for 45 s, annealing at 45 °C, 57 °C, 56 °C, 58 °C, 59 °C and 42 °C, according to primer specificity [7,14,43], for 45 s, an extension step at 72 °C for 45 s and a final extension step at 72 °C for 7 min.

#### 4.3.2. Detection of CRISPR Loci and CRISPR-Associated (Cas) Genes

The presence of three CRISPR loci (CRISPR1, CRISPR2 and CRISPR3) and the associated *cas* genes for CRISPR1 and CRISPR3 was detected by PCR, according to Huescas et al. [35]. The reactions conditions were as follows: initial denaturation at 94 °C for 10 min, followed by 30 cycles of denaturation at 94 °C for 45 s, annealing at 49 °C, 54 °C and 52 °C, according to primer specificity [35], for 45 s, an extension step at 72 °C for 45 s and a final extension step at 72 °C for 7 min.

#### 4.3.3. Identification of Mobile Genetic Elements (MGEs):

The presence of genes for inducible pheromones (*cpd*, *cob* and *ccf*), aggregation substances (*agg*, *asa1*, *prgB* and *asa373*), the Tn916–1545 transposon family (*Int-Tn*, the integrase gene) and the pheromone-responsive plasmid pCF10 (*prgW*, replication initiator protein gene), defining the ability of the isolates to transfer antibiotic resistance genes, was analyzed [7,44,45,46,47,48]. The reactions conditions were as follows: initial denaturation at 94 °C for 10 min, followed by 30 cycles of denaturation at 94 °C for 45 s, annealing at 54 °C, 52 °C, 50 °C, 61 °C, 53 °C and 55 °C, according to primer specificity [7,44,45,46,47,48], for 45 s, an extension step at 72 °C for 45 s and a final extension step at 72 °C for 7 min.

### 4.4. Genome Sequencing and Bioinformatic Analyses

The draft genomes of the selected strains (*E. faecalis* CA4, BM5, BM12 and BM15) were generated with Nanopor Minion Mk1B long-read. The genomes were assembled with Flye version 2.9.5 [49] in the Galaxy platform. The polishing of the sequences was obtained by Minimap version 2.2.8 [50], Racon version 1.5.0 [51] and Medaka version 1.7.2 [52]. The processed sequences were deposited at NCBI Genome, with approved accession numbers as follows: CP173761-CP173764 (*E. faecalis* CM4); CP173669-CP173671 (*E. faecalis* BM5); CP173666-CP173668 (*E. faecalis* BM12); CP173758-CP173760 (*E. faecalis* BM15). The circular and linear representations of the sequences were generated by CGView.js [53] in Proksee [54]. CARD (Comprehensive Antibiotic Resistance Database) in Proksee was used for the characterization of antibiotic resistance genes of the sequenced strains [55]. The identification of MGEs was carried out using MobileElementFinder v1.0.3 (2020-10-09) software. The investigation of the CRISPR systems, including the presence of CRISPR loci and *cas* genes, was carried out through analysis of the assembled genomes by CRISPRCasFinder 4.2.20 software [56]. CRISPRTarget with the json files from CRISPRCasFinder were used for identifying potential CRISPR targets [57]. For alignment of the spacers, Genbank-Phage, RefSeq-Plasmid and IMGVR databases were used (the default settings). The unidentified spacers were subjected to analysis by BLASTN in the NCBI database. Subtyping of the CRISPR repeats was carried out by RepeatTyper [58]. RNA secondary structures of the direct repeats were predicted by the RNAfold web server with default settings [59].

### 4.5. Statistical Analysis

Welch’s *t*-test was used to compare the number of genes for tetracycline resistance, genes for inducible pheromones and aggregation substances between enterococcal strains with different origins as well as between different enterococcal species. The number of CRISPR loci was also compared in the same manner. The results were considered significant when *p*  <  0.05.

## 5. Conclusions

In conclusion, this study mainly analyzes the relationship between CRISPR systems, mobile genetic elements and tetracycline resistance in different enterococcal species of non-clinical origin. The results found several connection points that indicate the presence of exogenous DNA in the form of plasmids, MGEs or tetracycline resistance genes in relation to inactive CRISPR systems. The analyzed spacers confirmed that plasmids target only one of the four sequenced strains, while in all of the CRISPR loci, self-targeted repeats were detected. These findings generally describe the acquisition of foreign DNA as a consequence of CRISPR inactivation, and self-targeting spacers as the main cause. Although the sequenced data in this study are limited, they will help to understand the evolutionary direction of the enterococcal population and lay the foundation for more thorough investigation of CRISPR systems in enterococcal species other than *E. faecalis* and *E. faecium*.

## 6. Strengths and Limitations

Although most studies on CRISPR systems and antibiotic resistance have focused primarily on clinical enterococcal isolates, our study confirms the need and importance of studying non-clinical ones. This is due to their significant ability to act as vectors of antibiotic resistance and virulence factors. In our work, we find primarily non-functional CRISPR systems in our isolates, suggesting the active participation of non-clinical enterococci in the HTG in the studied habitats. Moreover, the distribution of these adaptive systems and the immunity associated with them in bacteria are species-specific, even strain-specific, and largely dependent on the habitat.

In order to gain a larger picture of the relationship between the functionality of CRISPR systems and antibiotic resistance, whole-genome sequencing of a large number of strains is needed. This can be considered a limiting factor in many studies on the topic.

## Figures and Tables

**Table 1:** Comparison of the genotypic and phenotypic profile for antibiotic resistance to tetracycline for the enterococcal non-clinical isolates.

Presence of *tet* Genes	Number of Strains (n), %	Strains	Origin	Inhibition Zone (mm)	CLSI Interpretation
*tetM*	(n = 22), 31%	*E. gallinarum* BY17	Bulgarian yogurt	14	R
		*E. faecium* CM1	Cow milk	19	S
		*E. faecalis* CM4		15	I
		*E. faecalis* YFC1	Young feta cheese	14	R
		*E. faecalis* YFC3		28	S
		*E. pseudoavium* CA9	*Cornu aspersum*	14	R
		*E. pallens* CA10		16	I
		*E. faecalis* BM2	Breast milk	25	S
		*E. faecalis* BM3–BM9 *E. faecalis* BM14–BM16		14	R
		*E. faecalis* BM11, BM12		13	R
		*E. faecalis* BM10, BM13		16	I
*tetS*	(n = 6), 8%	*E. faecalis* YFC3	Young feta cheese	28	S
		*E. mundtii* CA8	*Cornu aspersum*	28	S
		*E. pallens* CA10		16	I
		*E. devriesei* CA13		49	S
		*E. devriesei* CA16		17	I
		*E. faecalis* BM15	Breast milk	14	R
*tetO*	(n = 2), 3%	*E. durans* YFC5	Young feta cheese	32	S
		*E. malodoratus* CA11	*Cornu aspersum*	50	S
*tetT*	(n = 1), 1%	*E. gallinarum* CA15	*Cornu aspersum*	35	S
*tetM+tetS*	(n = 3), 4%	*E. pallens* CA10	*Cornu aspersum*	16	I
		*E. faecalis* BM15	Breast milk	14	R
		*E. faecalis* YFC3	Young feta cheese	28	S

**Table 2:** Distribution of CRISPR loci and Cas proteins within the non-clinical enterococcal isolates.

Strains	*CRISPR*					Strains	*CRISPR*				
	*CRISPR1–cas csn1*	*CRISPR1–cas loci*	*CRISPR2 loci*	*CRISPR3–cas csn1*	*CRISPR3–cas loci*		*CRISPR1–cas csn1*	*CRISPR1–cas loci*	*CRISPR2 loci*	*CRISPR3–cas csn1*	*CRISPR3–cas loci*
*E. faecium* CM1						*E. faecalis* BY25					
*E. durans* CM2						*E. faecalis* BY26					
*E. durans* CM3						*E. faecalis* BY27					
*E. faecalis* CM4						*E. mundtii* CA1					
*E. faecalis* YFC1						*E. casseliflavus* CA2					
*E. durans* YFC2						*E. gilvus* CA3					
*E. faecalis* YFC3						*E. mundtii* CA4					
*E. durans* YFC4						*E. casseliflavus* CA5					
*E. durans* YFC5						*E. mundtii* CA6					
*E. faecium* MFC1						*E. mundtii* CA7					
*E. faecium* MFC2						*E. mundtii* CA8					
*E. faecium* DK1						*E. pseudoavium* CA9					
*E. faecium* BY1						*E. pallens* CA10					
*E. faecalis* BY2						*E. malodoratus* CA11					
*E. faecalis* BY3						*E. casseliflavus* CA12					
*E. faecalis* BY4						*E. devriesei* CA13					
*E. faecalis* BY5						*E. gallinarum* CA14					
*E. faecalis* BY6						*E. gallinarum* CA15					
*E. species* BY7						*E. devriesei* CA16					
*E. species* BY8						*E. mundtii* CA17					
*E. casseliflavus* BY9						*E. faecalis* BM1					
*E. faecalis* BY10						*E. faecalis* BM2					
*E. faecalis* BY11						*E. faecalis* BM3					
*E. faecium* BY12						*E. faecalis* BM4					
*E. faecium* BY13						*E. faecalis* BM5					
*E. faecium* BY14						*E. faecalis* BM6					
*E. faecium* BY15						*E. faecalis* BM7					
*E. faecium* BY16						*E. faecalis* BM8					
*E. gallinarum* BY17						*E. faecalis* BM9					
*E. casseliflavus* BY18						*E. faecalis* BM10					
*E. casseliflavus* BY19						*E. faecalis* BM11					
*E. casseliflavus* BY20						*E. faecalis* BM12					
*E. casseliflavus* BY21						*E. faecalis* BM13					
*E. faecalis* BY22						*E. faecalis* BM14					
*E. faecalis* BY23						*E. faecalis* BM15					
*E. faecalis* BY24						*E. faecalis* BM16					

pink color—negative result, no amplification product; green color—positive result, specific amplification product.

**Table 3:** Distribution of genes responsible for HGT within the non-clinical enterococcal isolates.

Strains	Genes Responsible for HGT									Strains	Genes Responsible for HGT								
	*cpd*	*cob*	*ccf*	*prgW*	*Int-tn*	*asa1*	*prgB*	*asa373*	agg		*cpd*	*cob*	*ccf*	*prgW*	*Int-tn*	*asa1*	*prgB*	*asa373*	agg
*E. faecium* CM1										*E. faecalis* BY25									
*E. durans* CM2										*E. faecalis* BY26									
*E. durans* CM3										*E. faecalis* BY27									
*E. faecalis* CM4										*E. mundtii* CA1									
*E. faecalis* YFC1										*E. casseliflavus* CA2									
*E. durans* YFC2										*E. gilvus* CA3									
*E. faecalis* YFC3										*E. mundtii* CA4									
*E. durans* YFC4										*E. casseliflavus* CA5									
*E. durans* YFC5										*E. mundtii* CA6									
*E. faecium* MFC1										*E. mundtii* CA7									
*E. faecium* MFC2										*E. mundtii* CA8									
*E. faecium* DK1										*E. pseudoavium* CA9									
*E. faecium* BY1										*E. pallens* CA10									
*E. faecalis* BY2										*E. maloduratus* CA11									
*E. faecalis* BY3										*E. casseliflavus* CA12									
*E. faecalis* BY4										*E. devriesei* CA13									
*E.faecalis* BY5										*E. gallinarum* CA14									
*E.faecalis* BY6										*E. gallinarum* CA15									
*E. species* BY7										*E. devriesei* CA16									
*E. species* BY8										*E. mundtii* CA17									
*E. casseliflavus* BY9										*E. faecalis* BM1									
*E. faecalis* BY10										*E. faecalis* BM2									
*E. faecalis* BY11										*E. faecalis* BM3									
*E. faecium* BY12										*E. faecalis* BM4									
*E. faecium* BY13										*E. faecalis* BM5									
*E. faecium* BY14										*E. faecalis* BM6									
*E. faecium* BY15										*E. faecalis* BM7									
*E. faecium* BY16										*E. faecalis* BM8									
*E. gallinarum* BY17										*E. faecalis* BM9									
*E. casseliflavus* BY18										*E. faecalis* BM10									
*E. casseliflavus* BY19										*E. faecalis* BM11									
*E. casseliflavus* BY20										*E. faecalis* BM12									
*E. casseliflavus* BY21										*E. faecalis* BM13									
*E. faecalis* BY22										*E. faecalis* BM14									
*E. faecalis* BY23										*E. faecalis* BM15									
*E. faecalis* BY24										*E. faecalis* BM16									

pink color—negative result, no amplification product; green color—positive result, specific amplification product.

**Table 4:** CRISPR repeats and spacer sequences in *E. faecalis* genomes.

Isolate	Repeat Sequence	CRISPR Repeat Subtyping	Secondary RNA Structures	* MFE [kcal/mol]	Spacer Sequence	Spacer Target
CM4	GTTTTAGAGTCATGTTGTTTAGAATGGTACCAAAAC	II-A	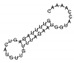	−2.30	GGTTATTATGTTACTGGTTACTTTAAAGAC	chromosome
					ATAATGATGTACAATTTATTCAAAACCATA	phage
					GAAAAGCAGTTCGAGCGGAAACTGCGACCA	phage
					GACTTACAAAAGACTGTGATTTACGTTATA	phage
					AAACTTTTTTGATTTGGCTTTTTCTCCCT	phage
	ACAAGGTGACCAAAGGGAACGTTGT	VI-B1	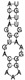	−7.10	CTCCTCTATGTTAAAACAAACTGCTTAGCCAAAAACATGGAGTAGATGATGAACAGC	phage
	ATAGTTGGCGAGCAACAGAAAAAC	III-B	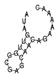	−3.20	TCGATATAGAATTGGACGTAGAGCCA	phage
BM5	GTTGGTTTTTCCCACTTTCGAACA	IV-A2	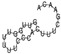	−1.40	AAGTACTGGTATTATTGGATTCTTCTGGAC	plasmid
					AAACGCCGATTTTATCATGTTTATCCGAAG	plasmid
	GTTTTGGTACCATTCTAAACAACATGACTCTAAAAC	II-A	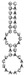	−3.40	TCTAATTTTTGAGTAATCGTACCAACTTGG	chromosome
					CTACGTCTTAACAAAGATAATTTAAAAGGT	phage
					GAAGCTACGTTTAAACCCGAAACCCCACTA	chromosome
					TAGGTAAGTAACTTAACCCTAGGTCAATCG	chromosome
	ATAGTTGGCGAGCAACAGAAAAAC	III-B	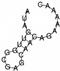	−3.20	TCGATATAGAATTGGACGTAGAGCCA	phage
BM 12	TAAAACAAACTGCTTAGCCAAAAACATGGAG	II-A	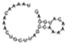	−1.60	TAGATGATGAACAGTACAAGGTGACCAAAGGGAACGTTGTCTCCTCTGTGC	phage
	GTTTTAGAGTCATGTTGTTTAGAATGGTACCAAAAC	II-A	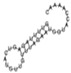	−2.30	AAGTACGGCATTACGCATTCCCCACTTTCT	phage
					GTAACAAACGATTAACTTTCGCATAGTCAT	phage
					AACCGAACTTACACCAACTGCGGATGGTAT	phage
					TATCGAAAATGATGTATTAATTTTAGGCTA	phage
					TACCTATGCAGACATTAAGAATTTACCAGA	phage
					TTATTTGAGAATCTGAAACATTTAGTTCAT	phage
					ATTTTGATGCATTAGCACCAAAATCAAAAG	phage
					ATTACTTGTTAAGGCTTCAATTATCAATTC	phage
					AAACTTTTTTGATTTGGCTTTTTCTCCCCT	chromosome
BM 15	GTTTTAGAGTCATGTTGTTTAGAATGGTACCAAAAC	II-A	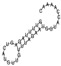	−2.30	TAAAGCAGCTTCTAAAACAGAAGGTGAAAT	phage
					GATTGGTAAGATTACATGATCTTTAGTACG	chromosome
					AAAGAAATGGACACATTACACAACGCTTTC	phage
					TAAAAACAAGACGAAATGAGGAAATTAACA	chromosome
					CAATGTAAATGCTCATTATGATTTACATAT	chromosome
	GTTTTGGTACCATTCTAAACAACATGACTCTAAAAC	II-A	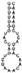	−3.40	AAATTTTTTGAACTTAATGCAATTTCTTGA	chromosome
					TTTGATAATCCAGAATCAACATCTTCACCA	phage
					TGCATAATAATCTTTTCTCTTAATGTTTTT	phage
					AACCCTCTTACTATGAGTTCCATTTATTTT	phage

* MFE—minimal free energy.

## Data Availability

The data presented in this research are available in the manuscript.

## Supplementary Materials

The following supporting information can be downloaded at https://www.mdpi.com/article/10.3390/antibiotics14020145/s1. Figure S1: Genome map of strain *E. faecalis* CM4, one chromosome and three plasmids. Red color—genes for antibiotic resistance, blue color—CRISPR systems. The image was generated with Map Builder in Proksee software, version 2.0.5. (https://proksee.ca/); Figure S2: Genome map of strain *E. faecalis* BM5, one chromosome and two plasmids. Red color—genes for antibiotic resistance, blue color—CRISPR systems. The image was generated with Map Builder in Proksee software, version 2.0.5. (https://proksee.ca/); Figure S3: Genome map of strain *E. faecalis* BM12, one chromosome and two plasmids. Red color—genes for antibiotic resistance, blue color—CRISPR systems. The image was generated with Map Builder in Proksee software, version 2.0.5. (https://proksee.ca/); Figure S4: Genome map of strain *E. faecalis* BM15, one chromosome and two plasmids. Red color—genes for antibiotic resistance, blue color—CRISPR systems. The image was generated with Map Builder in Proksee software, version 2.0.5. (https://proksee.ca/). Table S1 *Tet*-negative enterococcal strains and their phenotypic patterns to tetracycline.
