# Supplementary material for: Relationship Between CRISPR–Cas Systems and Acquisition of Tetracycline Resistance in Non-Clinical Enterococcus Populations in Bulgaria
Source: Antibiotics (Basel). 2025 Feb 2;14(2):145. doi: 10.3390/antibiotics14020145 (PMC11852239; doi:10.3390/antibiotics14020145)
Supplement: Supplementary file 1 [file antibiotics-14-00145-s001.zip › antibiotics-3409839-supplementary.pdf]

Table S1 *Tet*-negative enterococcal strains and their phenotypic petterns to tetracycline.

**Presence of *tet* genes**	**Number of strains (n), %**	**Strains**	**Origin**	**Inhibition zone (mm)**	**CLSI interpretation**
tet-negative strains	(n=44), 61%	*E. durans* CM2	Cow milk	29	S
		E. durans CM3		30	
		E. durans YFC2	Young feta cheese	30	
		E. durans YFC4		33	
		E. faecium MFC1	Matured feta cheese	37	
		E. faecium MFC2		35	
		E. faecium DK1	Doner kebap	35	
		E. faecium BY1, BY14; *E. faecalis* BY2, BY24; *E. casseliflavus* BY20	Bulgarian yogurt	35	
		E. faecalis BY3		36	
		E. faecalis BY4		39	
		E. faecalis BY5, BY23; *E. gallinarum* BY17		30	
		E. faecalis BY6; *E. faecium* BY13		33	
		E. sp. BY7		37	
		E. sp. BY8, *E. casseliflavus* BY9		14	R
		E. faecalis BY10, BY26; *E. faecium* BY12, BY15; *E. casseliflavus* BY18-BY19		34	S
		E. faecalis BY11		29	
		E. faecium BY16		32	
		E. casseliflavus BY21; *E. faecalis* BY22, BY25		31	
		E. mundtii CA1	*Cornu aspersum*	33	
		E. casseliflavus CA2		28	
		E. gilvus CA3		50	
		E. mundtii CA4, CA6, CA8		30	
		E. casseliflavus CA5		32	
		E. casseliflavus CA12		35	
		E. gallinarum CA14		37	
		E. mundtii CA17		34	
		E. faecalis BM1	Breast milk	27	

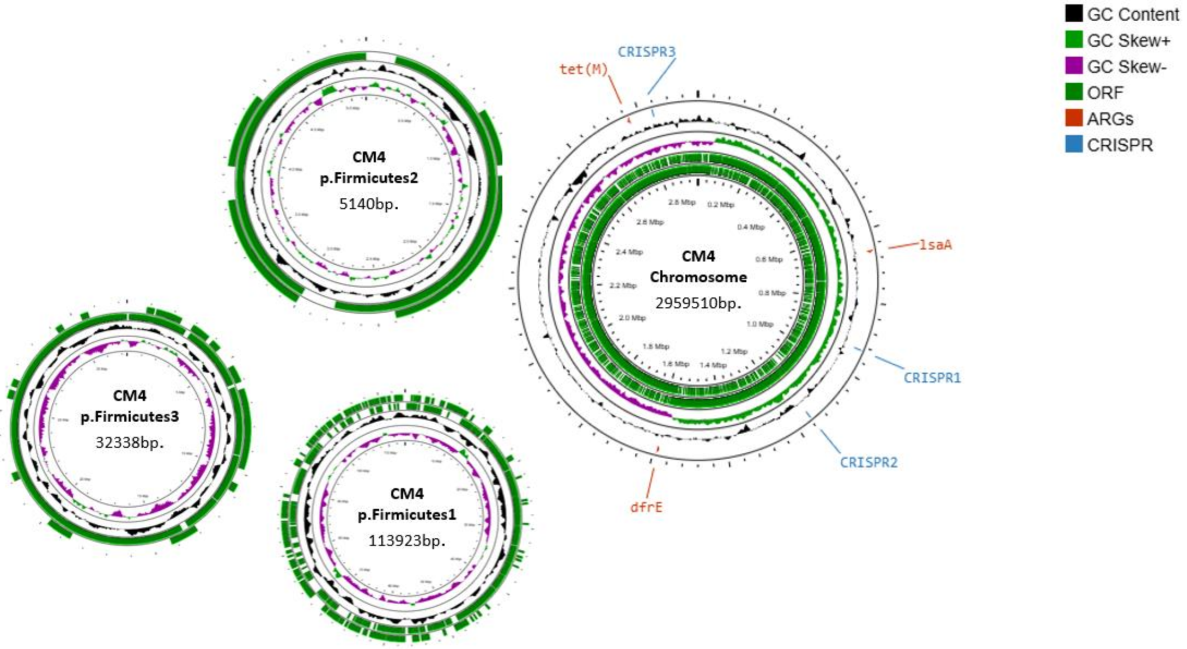

Figure S1: Genome map of strain *E. faecalis* CM4, one chromosome and three plasmids. Red color - genes for antibiotic resistance, blue color - CRISPR systems. The image was generated with Map Builder in Proksee software, version 2.0.5. (https://proksee.ca/).

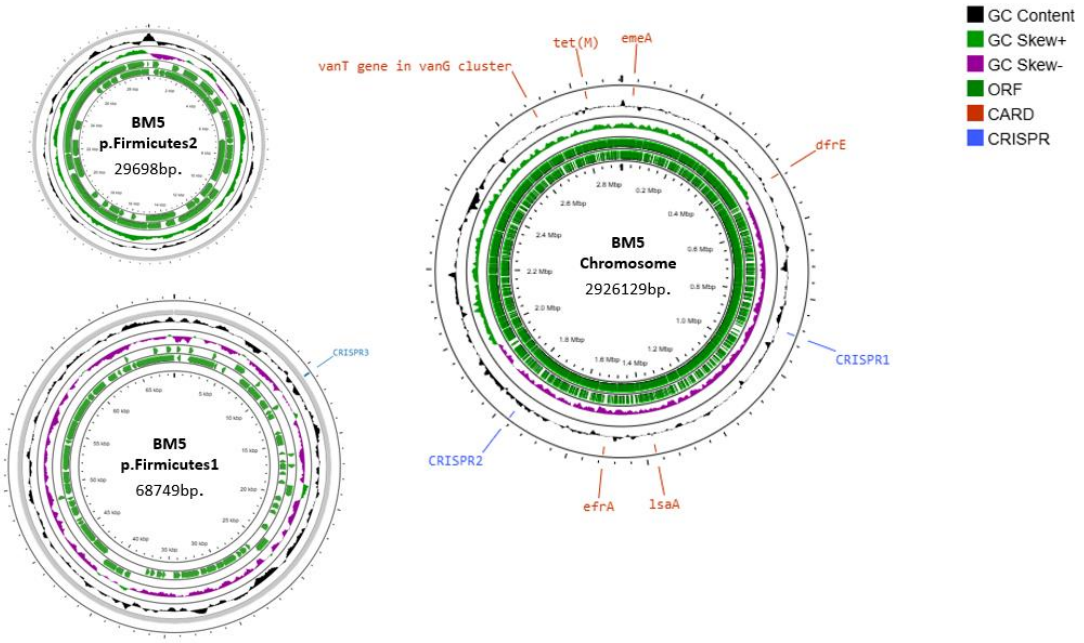

Figure S2: Genome map of strain *E. faecalis* BM5, one chromosome and two plasmids. Red color - genes for antibiotic resistance, blue color - CRISPR systems. The image was generated with Map Builder in Proksee software, version 2.0.5. (https://proksee.ca/).

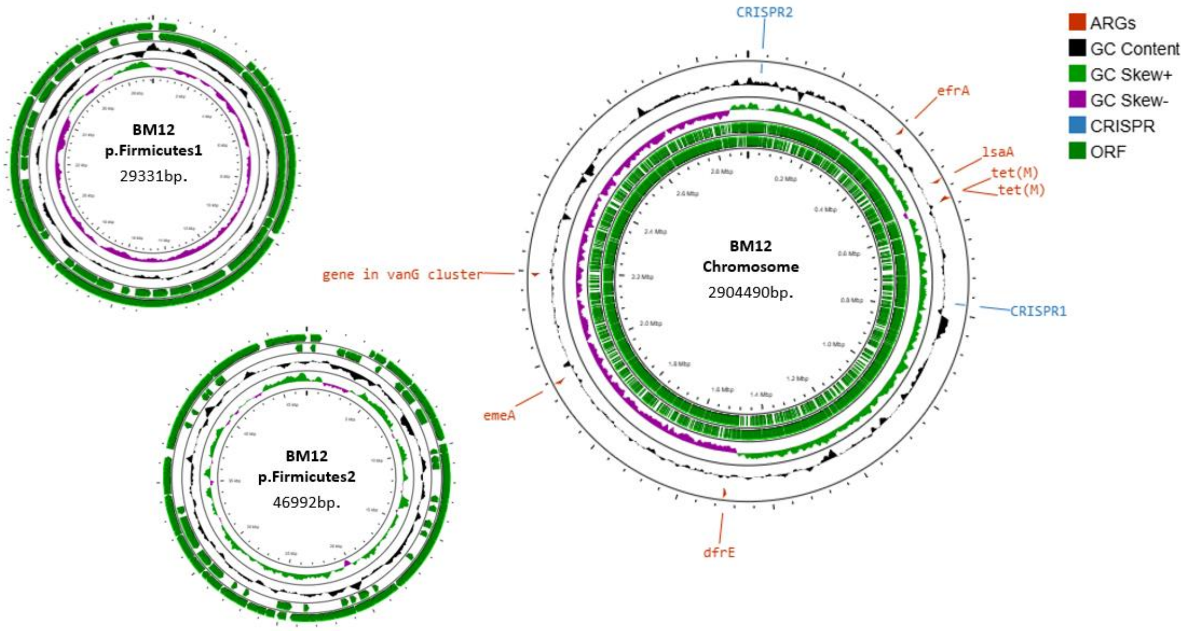

Figure S3: Genome map of strain *E. faecalis* BM12, one chromosome and two plasmids. Red color - genes for antibiotic resistance, blue color - CRISPR systems. The image was generated with Map Builder in Proksee software, version 2.0.5. (https://proksee.ca/).

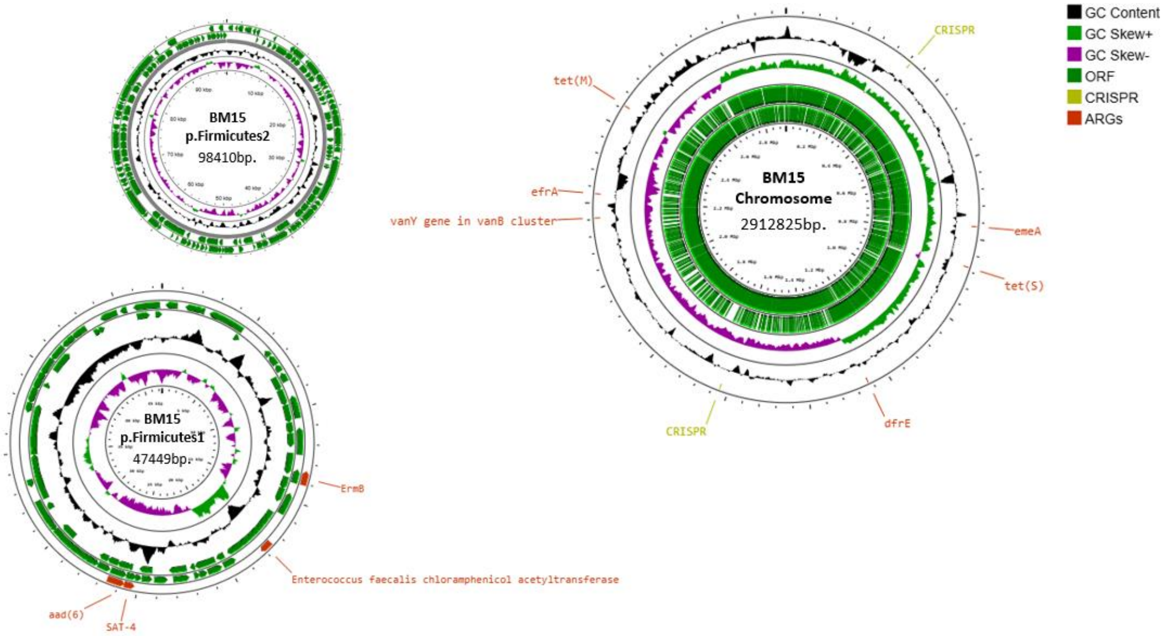

Figure S4: Genome map of strain *E. faecalis* BM15, one chromosome and two plasmids. Red color - genes for antibiotic resistance, blue color - CRISPR systems. The image was generated with Map Builder in Proksee software, version 2.0.5. (https://proksee.ca/).
